# PRDX2 Promotes the Proliferation and Metastasis of Non-Small Cell Lung Cancer *In Vitro* and *In Vivo*

**DOI:** 10.1155/2020/8359860

**Published:** 2020-08-27

**Authors:** Yun Chen, Sifu Yang, Hongying Zhou, Dan Su

**Affiliations:** Department of Oncology, Zhejiang Provincial People's Hospital, People's Hospital of Hangzhou Medical College, Hangzhou, China

## Abstract

**Purpose:**

Previous studies have reported that the levels of PRDX2 were correlated with tumorigenicity, recurrence, and prognosis of patients with different cancers. We investigated the association between PRDX2 levels and the prognosis of lung cancer patients. We also measured PRDX2 expression of non-small cell lung cancer (NSCLC) cells and examined its roles in the proliferation and migration *in vitro* and *in vivo*.

**Methods:**

We used the Kaplan–Meier plotter to analyze the survival of different levels of PRDX2 in lung cancer patients. The expression of PRDX2 in normal bronchial epithelial cell line and NSCLC cell lines was measured by qRT-PCR and western blot assays. Biological functions of NSCLC cells were detected by CCK8 and Transwell assays. We constructed tumor growth model using subcutaneously injection of nude mice and metastasis model by tail vein injection *in vivo*. The protein levels of proliferation related markers were measured by immunohistochemistry assay. Immunofluorescence method was used to detected EMT-related proteins.

**Results:**

The high levels of PRDX2 were associated with bad prognosis in lung cancer patients, especially in patients with adenocarcinoma. The expression of PRDX2 in NSCLC cell lines was higher than normal bronchial epithelial cells. Knockdown of PRDX2 inhibited the proliferation, migration, and invasion in A549 cells, while overexpression of PRDX2 promoted the malignancy in NCI-H1299 cells *in vitro*. Silencing PRDX2 restrained tumor growth and repressed lung metastasis by EMT *in vivo*.

**Conclusion:**

Our data indicates that PRDX2 functions as a protumor regulator and is involved in tumorigenesis and tumor progression of lung cancer.

## 1. Introduction

Lung cancer is the most frequently occurring malignancy in cancer patients among worldwide, with very high incidence and fatality rate [1]. Despite remarkable progresses have been made in the diagnosis and therapy of lung cancer, it is still a life-threatening disease with poor prognosis [2]. Lung cancer falls into two major classes, namely non-small cell lung cancer (NSCLC) and small cell lung cancer (SCLC). NSCLC is the most frequently specie of lung cancers and is usually diagnosed at an advanced stage with bad clinical outcomes [3]. Thus, it is extremely critical to find out the molecular mechanisms and novel therapeutic targets of lung cancer.

The increased reactive oxygen species (ROS) has powerful effects on intracellular signaling pathways in normal physiology and disease [4, 5]. Peroxiredoxins (PRDXs), belonging to a family of antioxidant peroxidases that scavenge ROS, also play vital roles in cell proliferation, tumorigenicities, cancer metastasis, and causes of drug resistance [6, 7]. PRDX2, as one member of Peroxiredoxins family, has been found to be highly expressed in different tumors and exerts the effects on the regulation of occurrence and development of cancers. Recently, increasing attentions have been paid to the association with PRDX2 and the progression of cancers. Lehtonen et al. first showed that PRDX2 levels were significantly increased in lung cancer [8]. PRDX2 was significantly downregulated in melanoma, and the decreased expression resulted in inhibiting proliferation and migration of melanoma cells [9]. Similarly, there was also a significant reduction of the expression of PRDX2 in acute myeloid leukemia (AML) [10]. On the contrary, PRDX2 acted as an oncogene in many malignant tumors, including prostate cancer, gastric cancer, esophageal carcinoma, and cervical cancer [11–14]. Furthermore, a recent study showed that PRDX2 was related to resistance to 5-Fluorouracil (5-FU), one of the most common chemotherapeutic agents, and the reduced levels of PRDX2 could contribute to increasing the sensitivity of 5-FU in colon cancer cells [15]. Not only is it resistant to chemotherapeutic agents, PRDX2 also shows resistance to radiation therapy [16]. Moreover, it has also been reported that PRDX2 was associated with the maintenance of cancer stem cells (CSCs) in liver cancer [17]. However, the correlation between the levels of PRDX2 and the initiation of lung cancer has not been assessed yet.

The purpose of our study is to confirm the roles of PRDX2 in the progression of NSCLC and to elucidate whether PRDX2 affects the biological functions and tumourigenesis of NSCLC cells in order to provide a novel perspective therapeutic target of PRDX2 in NSCLC.

## 2. Materials and Methods

### 2.1. The Kaplan–Meier Plotter of Lung Cancer Patients

The Kaplan–Meier plotter (http://kmplot.com/analysis/) is accessible via the Internet, which has survival information for more than 54,000 genes in 21 cancer types, including breast, ovarian, lung, and gastric cancer. The system includes data sources from genetic microarrays and RNA-seq, and databases include Gene Expression Omnibus (GEO), The European Genome-phenome Archive (EGA), and The Cancer Genome Atlas (TCGA). The database has the survival information from 1,927 lung cancer patients, which includes clinical data such as gender, pathological classifications, clinical stage, grade, smoking history, chemotherapy, and radiotherapy [18]. In this study, we explored the relationship between the expression levels of PRDX2 and prognosis (including overall survival (OS) and progression-free survival (PFS)) in all lung cancer patients using the Kaplan–Meier Plotter analysis. The Affymetrix ID is valid: 201006_at (PRDX2). The expression of PRDX2 is split into “low” and “high” based on the median levels of genes from lung cancer samples. The package “survival” is used to calculate and plot the Kaplan–Meier survival curves. The number of cases and the risk numbers are indicated below the images. Hazard ratio, 95% confidence intervals, and logrank *P* value are calculated and displayed in the main images.

### 2.2. Cell Culture

All cell lines were obtained from the Cell Bank of Chinese Academy of Sciences (Shanghai, China). The human normal bronchial epithelial cell line BEAS-2B cells were cultured in Minimum Essential Media (MEM) (Gibco) supplemented with 10% fetal bovine serum (FBS) (Hyclone) and 1% penicillin and streptomycin (Beyotime Biotechnology, China). NSCLC cell lines NCI-H460, NCI-H1650, NCI-H1299, and A549 were cultured in Dulbecco's modified Eagle's medium (DMEM) (Gibco) supplemented with 10% FBS (Hyclone) and 1% penicillin and streptomycin (Beyotime Biotechnology, China). All cells were cultured in at 37°C with 5% CO_2_.

### 2.3. Plasmid Construction and Transfection

PRDX2 shRNAs and scrambled control shRNA were purchased from GenePharma Company (Shanghai, China). Using a Lipofectamine 2000 (Invitrogen)-based transfection method, A549 cells were transfected with PRDX2 shRNAs or scrambled control shRNA and selected with puromycin for 3-4 weeks. Specifically, 293T cells (30% confluence) were cultured in 6-well plates and transfected with PRDX2 shRNAs and scrambled control shRNA for 48 hours. Then, the medium from 293T cells was collected and was added to A549 cells (30%-50% confluence) for 48 h, followed by selection with 6 *μ*g/ml puromycin for 3-4 weeks. Plasmids (pcDNA3.1-PRDX2 and pcDNA3.1) were obtained from OBiO Technology Co. Ltd. (Shanghai, China). NCI-H1299 cells (70% confluence) were cultured in 6-well plates and transfected with pcDNA3.1-PRDX2 or pcDNA3.1 for 72 hours using Lipofectamine 2000.

### 2.4. Cell Proliferation

CCK8 assay (Dojindo, Japan) was used to measure cell proliferation. 2000-5000 cells were cultured in a 96-well plate, and then after overnight incubation, the cells were continuously cultured for 24 hours, 48 hours, and 72 hours. Then, after incubation, 10 *μ*l CCK8 reagent was added into each well of 96-well plates and incubated for 1 hour. After incubation of CCK8 solution, we measured the OD value at 450 nm.

### 2.5. Transwell Assays

Transwell assays were used to check the invasion and migration abilities of A549 and NCI-H1299 cells. For invasion assay, 60-80 *μ*l of Matrigel gel (BD Biosciences) was added into the upper chambers (Millipore) and incubated at 37°C in 5% CO_2_ for 2-4 hours. Then, 600 *μ*l complete medium was added in the 24-well plates and (5–10) × 10^4^ NCI-H1299 and A549 cells suspended with 200 *μ*l DMEM medium (serum-free) were seeded on the upper chambers and incubated for 24 hours. For migration assay, (1, 2) × 10^5^ NCI-H1299 and A549 cells were suspended with 200 *μ*l DMEM medium (serum-free) and then seeded on the upper chambers and incubated for 48 hours. After incubation, NCI-H1299 and A549 cells moved to the bottom of the 24-well chamber, followed fixing with 4% formaldehyde and dyeing with 0.1% crystal violet solution.

### 2.6. Quantitative Real-Time Polymerase Chain Reaction (RT-PCR)

Total RNA was extracted from cells using a TRIzol reagent (Invitrogen). One microgram RNA was used for reverse transcription (Takara, China) to generate cDNA, and RT-PCR was performed using SYBR Green reagents (Takara, China), followed cycling conditions: 95°C for 10 min, followed by 40 cycles at 95°C for 15 sec, 60°C for 10 sec, and 72°C for 60 sec. Relative PRDX2 expression was calculated using the 2^−*ΔΔ*CT^ method, and GAPDH was used as control. Primers are as follows: PCR PRDX2: 5′-CACCTGGCTTGGATCAACACC-3′ (forward), 5′-CAGCACGCCGTAATCCTCAG-3′ (reverse); GAPDH: 5′-TGACTTCAACAGCGACACCCA-3′ (forward), 5′-ACCCTGTTGCTGTAGCCAAA-3 (reverse).

### 2.7. Western Blot Assay

Protein levels were performed using western blotting assay. Protein was collected using RIPA lysis reagent (Beyotime Biotechnology, China) with protease inhibition (Thermo Fisher, USA), and the concentrations of protein were measured using a BSA kit (Thermo Fisher, USA). Equal amounts of 30 *μ*g protein were added into each lane, followed separating through 10% gels and transferring to PVDF membranes (Millipore, USA). After blocking with 5% milk, membranes were incubated with primary antibodies (anti-PRDX2, Abcam, ab109367, 1 : 1000 dilution; anti-*β*-actin, Bioworld, BS6007MH, 1 : 5000 dilution) overnight at 4°C, followed incubating with secondary antibodies (Cell Signaling Technology, 1 : 5000 dilution) for 2 hours at room temperature.

### 2.8. Animal Experiments

Six-week-old male SCID mice were obtained from Beijing Vital River Laboratory Animal Technology Co., Ltd. For primary tumors, PRDX2-knockdown or control A549 cells (5 × 10^6^ cells in 50 *μ*l DMEM medium) were subcutaneously injected into left axilla of 5 SCID mice, respectively. The tumor volume was calculated (volume = length × (width)^2^/2). Tumor volume and body weights of mice were recorded every three days. After 30 days, all mice were sacrificed and tumors were collected. For metastasis model, PRDX2-knockdown or control A549 cells (2 × 10^5^ cells in 50 *μ*l PBS) were injected into tail vein of 5 nude mice, respectively. After 2 months, all mice were sacrificed, and the lungs of the mice were collected. The animal experiments were performed following the Animal Care Committee of Zhejiang Provincial People's Hospital.

### 2.9. Hematoxylin and Eosin (H&E) and Immunohistochemistry

The tissues were fixed with 4% formaldehyde for 24 hours and embedded with paraffin. Sections (4 *μ*m) were deparaffinized in xylenes and hydrated through graded alcohols. The slides were stained with hematoxylin and eosin. For immunohistochemistry assay, the slides were boiled in antigen unmasking solution (pH 6.0) for 20 min and incubated with 0.3% H_2_O_2_ for 20 min. After blocking with 5% BSA, the slides were incubated with primary antibodies (anti-Ki-67, Abcam, ab15580, 1 : 200 dilution; anti-PCNA (Proliferating Cell Nuclear Antigen), Dako, M0879, 1 : 50 dilution) overnight at 4°C. The next day, incubation with secondary antibodies was performed at 37°C for 1 hour, followed treating with DAB chromogen.

### 2.10. Immunofluorescence

Tissues were embedded in paraffin. Sections were deparaffinized in xylenes and hydrated through graded alcohols. The sections were boiled in antigen unmasking solution (pH 6.0) for 20 min. After blocking with 5% BSA, the slides were incubated with primary antibodies (anti-E-cadherin, CST, #14472, 1 : 100 dilution; anti-Vimentin, CST, #5741, 1 : 100 dilution; anti-Slug, CST, #9585, 1 : 400 dilution) and DAPI (1 : 1000 dilution, Beyotime Biotechnology) overnight at 4°C. The next day, incubation with secondary antibodies was performed at 37°C for 1 hour.

### 2.11. Statistical Analysis

GraphPad Prism 7.0 was used to make all graphs and statistical analyses. The two-tailed Student's *t*-test and logrank test were used to performed statistical analysis. Student's *t*-test was used to analyze the statistical significance among two groups. One-way ANOVA was used to compare the differences among three or more groups. *P* value < 0.05 was judged to indicate statistical significance.

## 3. Results

### 3.1. Increased PRDX2 Is Related to Poor Prognosis of Lung Cancer Patients

We first investigated the relationship between the expression of PRDXs and the overall survival (OS) in patients with lung cancer by the Kaplan–Meier plotter (KMplot, http://kmplot.com/analysis/). The mRNA levels of PRDX1, PRDX2, PRDX3, PRDX5, and PRDX6 showed an excellent correlation with the OS of lung cancer patients (Table 1). However, only PRDX2, PRDX3, and PRDX6 expression was associated with worse OS in adenocarcinoma patients (Table 1). Moreover, PRDX2 and PRDX6 exhibited stronger connections than PRDX3 in adenocarcinoma patients (Table 1).

**Figure 1:**
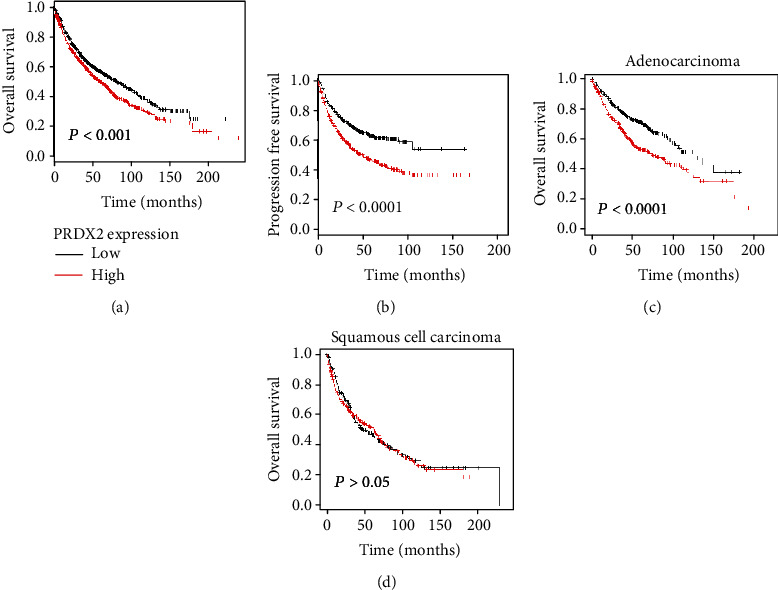
Increased PRDX2 is related to poor prognosis of lung cancer patients. The relationship between the expression of PRDX2 and overall survival (OS) (a) and relapse-free survival (PFS) (b) was performed by the Kaplan–Meier plotter (KMplot, http://kmplot.com/analysis/). (c, d) The association between the expression of PRDX2 and overall survival (OS) in adenocarcinoma patients or squamous cell carcinoma patients was performed by the Kaplan–Meier plotter.

Next, to better assess the role of PRDX2 played on clinical relevance in patients with lung cancer, we investigated the relationship between the levels of PRDX2 and the survival of lung cancer patients. The results suggested that higher PRDX2 expression was significantly correlated with worse OS (*P* < 0.001), in 1925 lung cancer patients (967 samples with low PRDX2 expression and 958 samples with high PRDX2 expression) (Figure 1(a)). Similarly, the increased expression of PRDX2 was remarkably associated with progression-free survival (PFS) (*P* < 0.0001), in 982 patients with lung cancer (491 samples with low PRDX2 expression and 491 samples with high PRDX2 expression) (Figure 1(b)). Furthermore, the high levels of PRDX2 were also related to bad OS in 719 adenocarcinoma patients (*P* < 0.0001) (363 samples with low PRDX2 expression and 356 samples with high PRDX2 expression) (Figure 1(c)). However, there was no significant difference in 524 squamous cell carcinoma patients (*P* > 0.05) (Figure 1(d)). These results suggest that higher levels of PRDX2 are associated with worse prognosis in lung cancer patients.

### 3.2. PRDX2 Is Significantly Upregulated in NSCLC Cells

**Figure 2:**
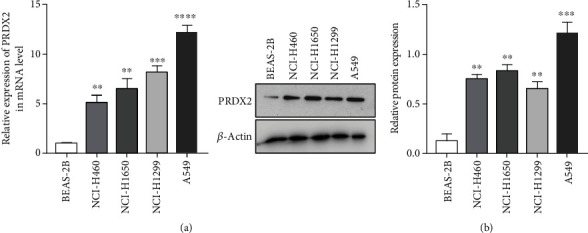
PRDX2 is significantly upregulated in NSCLC cells. The mRNA levels and protein levels of PRDX2 in human normal bronchial epithelial cell line (BEAS-2B) and various NSCLC cell lines (NCI-H460, NCI-H1650, NCI-H1299, and A549) were performed by qRT-PCR (a) and western blot assays (b). ∗∗*P* < 0.01; ∗∗∗*P* < 0.001; ∗∗∗∗*P* < 0.0001.

To further examine the potential effects of PRDX2 in lung cancer cells, we analyzed the expression levels of PRDX2 in human normal bronchial epithelial cell line (BEAS-2B) and commonly used NSCLC cell lines (NCI-H460, NCI-H1650, NCI-H1299, and A549). RT-PCR analysis showed that the mRNA levels of PRDX2 were obviously higher in NSCLC cells than in normal cells. As shown in Figure 2(a), compared with BEAS-2B cells, the expression of PRDX2 in NCI-H1299, NCI-H460, NCI-H1650, and A549 cells was 5.12 ± 0.73, 6.51 ± 0.99, 8.21 ± 0.61, and 12.20 ± 0.71 times higher, respectively (*P* < 0.01). Western blotting assay also investigated that the protein levels of PRDX2 in NSCLC cell lines (NCI-H460, NCI-H1650, NCI-H1299, and A549) were higher than BEAS-2B cells (Figure 2(b)). Among all NSCLC cells, PRDX2 expression was highest in A549 cells and lowest in NCI-H1299 cells.

### 3.3. Knockdown of PRDX2 Inhibits Proliferation, Migration, and Invasion in A549 Cells

**Figure 3:**
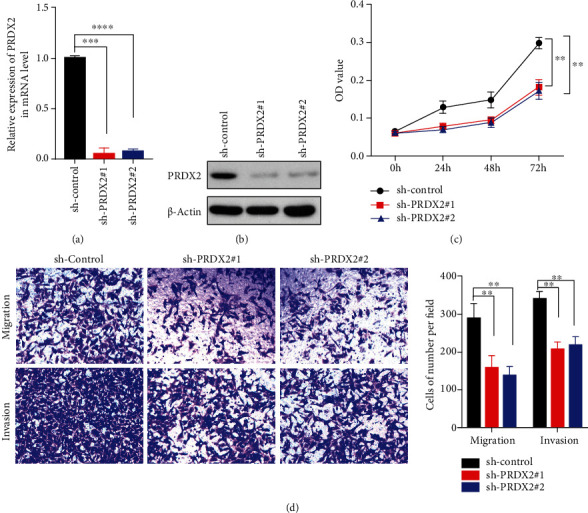
Knockdown of PRDX2 inhibits proliferation, migration, and invasion in A549 cells. (a) A549 cells were transfected with scrambled control shRNA or two PRDX2 shRNAs and followed using the qRT-PCR assay to measure the mRNA levels of PRDX2. (b) Western blot assay was used to check the protein levels of PRDX2. (c) Cell viability was performed by CCK8 assay. (d) Migration assay was performed using Transwell assay without Matrigel gel, and invasion assay was performed using Transwell assay with Matrigel gel. ∗∗*P* < 0.01; ∗∗∗*P* < 0.001; ∗∗∗∗*P* < 0.0001.

We have known that the expression of PRDX2 is upregulated in NSCLC cell lines, and there is an association between high levels of PRDX2 and bad clinical prognosis. To investigate whether the expression of PRDX2 is also related to biological function, we first knocked down the expression of PRDX2 using two shRNAs in A549 cells. After transfection and selection, the transfection efficiency showed silencing PRDX2 expression in A549 cells remarkably decreased both the mRNA levels and protein levels of PRDX2 using qRT-PCR and western blotting assays (Figures 3(a) and 3(b)).

Next, a proliferation assay was performed to determine whether PRDX2 depletion would affect the proliferation ability in A549 cells. As presented in (Figure 3(c)), cell viability was obviously reduced in sh-PRDX2 groups compared with sh-control groups by CCK8 assays. Furthermore, Transwell assays illustrated that the numbers of migrating and invasive cells in sh-PRDX2 groups were extremely less than that in sh-control group (Figure 3(d)). These data revealed that silencing PRDX2 expression inhibited proliferation, migration, and invasion in A549 cells.

### 3.4. Overexpression of PRDX2 Promotes Proliferation, Migration, and Invasion in NCI-H1299 Cells

**Figure 4:**
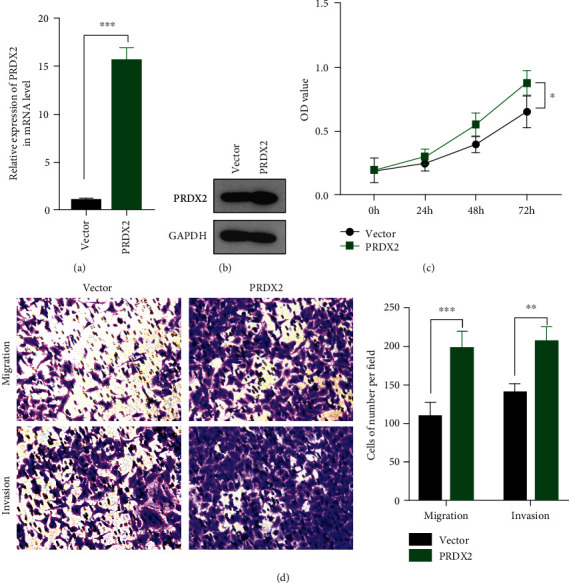
Overexpression of PRDX2 promotes proliferation, migration, and invasion in NCI-H1299 cells. (a) NCI-H1299 cells were transfected with PRDX2-overexpressed or controlled plasmids, and the mRNA levels of PRDX2 were performed by qRT-PCR assay. (b) The protein levels of PRDX2 were performed by western blot assay. (c) Cell viability was performed using CCK8 assay. (d) Migration assay was performed using Transwell assay without Matrigel gel, and invasion assay was performed using Transwell assay with Matrigel gel. ∗*P* < 0.05, ∗∗*P* < 0.01; ∗∗∗*P* < 0.001.

Since silencing PRDX2 expression repressed the malignancy of lung cancer, we next transfected NCI-H1299 cells with PRDX2 overexpressed or controlled plasmids for 72 hours. The results revealed that the expression of PRDX2 increased in PRDX2 overexpression group compared with vector group using qRT-PCR and western blotting assays (Figures 4(a) and 4(b)). Contrary to the results presented above, overexpression of PRDX2 increased the proliferation of NCI-H1299 cells in PRDX2 overexpressed cells compared with the control cells (*P* < 0.05; Figure 4(c)). In addition to its role in mediating the proliferation, overexpression of PRDX2 had a promoting role of migration and invasion abilities by Transwell assays (Figure 4(d)). Therefore, overexpression of PRDX2 can promote the viability, migration, and invasion of NSCLC cells *in vitro*.

### 3.5. Silencing PRDX2 Inhibits NSCLC Cell Proliferation *In Vivo*

**Figure 5:**
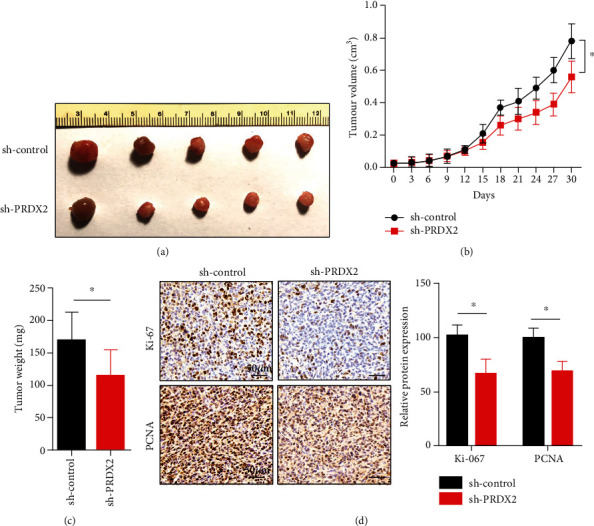
Silencing PRDX2 inhibits NSCLC cell proliferation *in vivo*. (a) PRDX2-knockdown or control A549 cells were subcutaneously injected into left axilla. The tumor volume (b) and tumor weights (c) in control group and PRDX2-knockdown were calculated. (d) The expression of Ki-67 and PCNA in control group and PRDX2-knockdown group was performed by immunohistochemistry assay. Scale bar = 50 *μ*m in (d). ∗*P* < 0.05.

To extend the *in vitro* findings concerning the influence of PRDX2 on mediating the proliferation, we assess whether PRDX2 could regulate cell growth *in vivo*. To do this, we stably knocked down PRDX2 in A549 cells and subcutaneously injected PRDX2-knockdown or control A549 cells into the SCID mice. The protein expression of PRDX2 was significantly reduced in the sh-PRDX2 group compared with the sh-control group by immunohistochemistry assay *in vivo* (Figure S1). As expected, tumors in PRDX2-depleted group showed a remarkable shrinkage, with significantly decreased tumor sizes and tumor weights at the end of experiment compared with the control group (Figures 5(a)–5(c)). Next, to investigate PRDX2's role in promoting cell proliferation *in vivo*, we performed immunohistochemistry assay to measure the protein levels of Ki-67 and proliferating cell nuclear antigen (PCNA) which were the most common proliferation related markers. The results presented that a marked decrease in the numbers of Ki-67-positive (Ki-67+) cells and PCNA-positive (PCNA+) cells was observed in PRDX2-knockdown tumor tissues (Figure 5(d)), indicating PRDX2 knockdown suppressed cell proliferation *in vivo*.

### 3.6. Knocking Down PRDX2 Suppresses NSCLC Cell Metastasis by EMT *In Vivo*

**Figure 6:**
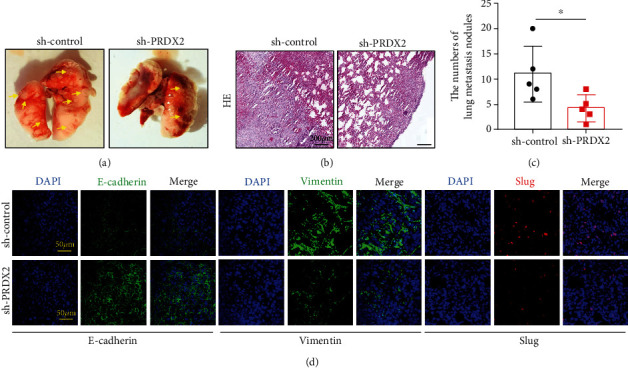
Knocking down PRDX2 suppresses NSCLC cell metastasis by EMT *in vivo.* (a) PRDX2-knockdown or control A549 cells were injected into tail vein. (b) HE staining of lung metastatic nodules of mice was performed. (c) The number of lung metastatic nodules of mice was calculated. (d) The expression of E-cadherin, Vimentin, and Slug in the control group and PRDX2-knockdown group was performed by immunofluorescence assay. Scale bar = 200 *μ*m in (b), scale bar = 50 *μ*m in (d), ∗*P* < 0.05, ∗∗*P* < 0.01; ∗∗∗*P* < 0.001.

Based on the observations of the functions of PRDX2 in migration and invasion of NSCLC cells, we next evaluated whether PRDX2 could also induce metastasis *in vivo.* We tested metastasis behavior changes using PRDX2-depleted or controlled A549 cells tail vein injection. There is a dramatic decrease in the numbers of pulmonary metastasis nodules in the PRDX2 knockdown group compared with control group (Figures 6(a)–6(c)).

Epithelial-mesenchymal transition (EMT), the important compositions of tumor progression, plays an indispensable role in increasing the migratory and invasive capacities in various types of cancers [19–21]. Therefore, suppressing EMT is a necessary method to inhibit tumor metastasis. To confirm whether the PRDX2-induced metastasis was dependent on EMT, we checked EMT-related protein levels using immunofluorescence method. Here, we showed that PRDX2-depleted lung metastasis tissues had obvious upregulation of E-cadherin expression and downregulation of Vimentin expression in protein levels compared with the controlled tissues (Figure 6(d)). Slug, an essential ingredient in EMT, could promote initiation of EMT by inhibiting the levels of E-cadherin [22]. Obviously, immunofluorescence results showed that silencing PRDX2 expression was significantly decreased Slug activity in lung metastasis tissues (Figure 6(d)). These data indicated that PRDX2 depletion could restrain metastasis via EMT in the NSCLC model.

## 4. Discussion

Peroxiredoxins (PRDXs) belong to a very ubiquitous family and already have six subtypes (PRDX1, PRDX2, PRDX3, PRDX4, PRDX5, and PRDX6) in mammals [23]. Additionally, depending on the number of cysteine remains, they are classified into two categories: 1-Cys and 2-Cys, of which PRDX1-PRDX5 belong to the 2-Cys subtype, while PRDX6 is the 1-Cys subtype [23, 24]. The aberrant expression of PRDXs has been shown in various tumors and is associated with tumorigenicity, prognosis, and recurrence of patients with different cancers [25–29]. Recently, several studies have shown that PRDXs are involved in the occurrence and development of breast cancer. Wang et al. discovered that the expression of PRDX1-PRDX5 was significantly increased, whereas the expression of PRDX6 was obviously reduced in human breast tissues compared with normal tissues [30]. Another study also revealed that higher mRNA expression of PRDX1/2/4/5/6 was correlated with worse prognosis, while high levels of PRDX3 were associated with favorable prognosis of patients with breast cancer [31].

Growing evidences indicate that there is a closely association between the PRDX2 expression and the progression of cancers. Previous studies have shown that PRDX2 expression is upregulated in different types of tumors such as gastric cancer and esophageal carcinoma. A recent study showed that PRDX2 was significantly increased in tissues and cell lines, and the overexpression of PRDX2 was associated with the poor clinical outcomes of gastric cancer [11]. Feng et al. demonstrated that PRDX2 acted as an oncogene in esophageal carcinoma (ESCA) and promoted the progression of ESCA [12]. PRDX2 promoted tumor angiogenesis via activating VEGFR2 in colorectal cancer [32]. There is now increasing evidence that PRDX2 expression was significantly increased and may become a prognostic and therapeutic target in lung cancer [8, 33, 34]. These data suggested that PRDX2 is crucial for the development of tumorigenesis and tumor progression.

As a broad and powerful regulator, PRDX2 may modulate essential factors and induce vital pathways in mammals. It has been shown that PRDX2 downregulated the expression of Dynamin 3 (DNM3), which is one member of the guanylate triphosphatases (GTPases) family and exerts as an important tumor suppressor [35]. A recent study reported that PRDX2 plays roles in the stem cell maintaining via activating the Hedgehog pathway in colon cancer [36]. Also, PRDX2 acts as a positive regulator through activation of Wnt/*β*-catenin and AKT signaling pathways in ESCA [12].

Herein, we hypothesize that PRDX2 may have powerful effects on the tumor progression of lung cancer. Here, the high levels of PRDX2 were associated with poor OS and PFS of patients with lung cancer. This situation happened in adenocarcinoma patients but not in squamous cell carcinoma patients. To investigate the biological function of PRDX2 in lung cancer, we knocked down PRDX2 using two shRNAs and reveled that silencing PRDX2 expression inhibited cell proliferation, migration, and invasion in NSCLC cell line A549 cells. Contrarily, overexpression of PRDX2 enhanced the malignancy of NSCLC cell line NCI-H1299 cells. In order to test the influences of PRDX2 expression in the more complex *in vivo* environment, we examined the NSCLC cell tumorigenicity by xenograft model and found that knockdown PRDX2 weakened the tumor formation *in vivo*. In addition, lower PRDX2 expression was associated with less pulmonary metastasis nodules, suggesting that PRDX2 might promote the metastasis of lung cancer. However, there are some limitations and shortcomings in this study: we did not investigate the correlation of PRDX2 expression and tumor growth or metastasis of lung cancer patients. We will collect tissues of lung cancer patients and confirm this results in the future.

Metastasis is the main cause of the treatment failure and death in lung cancer [37], and there has been extensive research aimed at understanding the mechanism of metastasis. The EMT process is the molecular basis of cancer metastasis, and the measurement of EMT is widely used in tumor metastasis [38, 39]. Slug is a critical and essential EMT modulator and initiates EMT by inhibiting the expression of E-cadherin [22]. A recent study reported that miR-200b-3p is a direct posttranscriptional regulator of PRDX2, which suppresses the malignant behaviors of colorectal cancer (CRC) cells through inhibition of PRDX2 [40]. Downregulation of miR-200 enhances the proliferation and metastasis of cancer cells and also regulates EMT process [5, 41, 42]. Thus, we believe that PRDX2 may promote the metastasis by EMT in NSCLC. In this study, immunofluorescence results showed that silencing PRDX2 expression was significantly decreased Vimentin expression and Slug activity while increased E-cadherin expression in lung metastasis tissues compared with the control tissues *in vivo*. The data indicated that knockdown of PRDX2 may inhibit metastasis though EMT in NSCLC animal model.

## 5. Conclusions

This study showed that PRDX2 is highly expressed in lung cancer, and it is correlated with worse OS and PFS of lung cancer patients. PRDX2 plays important roles in the development of tumorigenesis and tumor progression of non-small cell lung cancer. PRDX2 may potentially become a novel therapeutic target for lung cancer.

## Figures and Tables

**Table 1:** Association analysis between PRDX mRNA expression and histology of the OS in patients with lung cancer.

Gene	Affymetrix ID	Histology	HR (95% CI)	Logrank *P*
PRDX1	208680_at	All	1.33 (1.17-1.51)	1e-05∗∗∗∗
		Ade	1.09 (0.87-1.38)	0.46
		SCC	1.22 (0.97-1.55)	0.092
PRDX2	201006_at	All	1.25 (1.1-1.42)	0.00044∗∗∗
		Ade	1.64 (1.3-2.08)	3e-05∗∗∗∗
		SCC	1.03 (0.82-1.31)	0.78
PRDX3	209766_at	All	1.23 (1.09-1.4)	0.0012∗∗
		Ade	1.38 (1.1-1.75)	0.0058∗∗
		SCC	1.1 (0.87-1.4)	0.43
PRDX4	201923_at	All	0.99 (0.87-1.12)	0.85
		Ade	0.93 (0.74-1.17)	0.55
		SCC	0.79 (0.62-1.01)	0.055
PRDX5	222994_at	All	0.81 (0.69-0.95)	0.011∗
		Ade	0.97 (0.76-1.23)	0.8
		SCC	0.8 (0.59-1.09)	0.15
PRDX6	200844_s_at	All	1.29 (1.14-1.46)	8.7e-05∗∗∗∗
		Ade	1.75 (1.37-2.24)	5e-06∗∗∗∗
		SCC	1.16 (0.92-1.47)	0.22

All: all patients; Ade: adenocarcinoma; SCC: squamous cell carcinoma; ∗*P* < 0.05; ∗∗*P* < 0.01; ∗∗∗*P* < 0.001; ∗∗∗∗*P* < 0.0001.

## Data Availability

The data used to support the findings of this study are available from the corresponding author upon request.

## Supplementary Materials

Supplementary Materials Figure S1: the expression of PRDX2 after knocking down PRDX2 *in vivo*. The protein expression of PRDX2 was significantly reduced in the sh-PRDX2 group compared with the sh-Control group by immunohistochemistry assay *in vivo*.
