# Supplementary figures and images for: PRDX2 Promotes the Proliferation and Metastasis of Non-Small Cell Lung Cancer In Vitro and In Vivo
Source: Biomed Res Int. 2020 Aug 27;2020:8359860. doi: 10.1155/2020/8359860 (PMC7474358; doi:10.1155/2020/8359860)

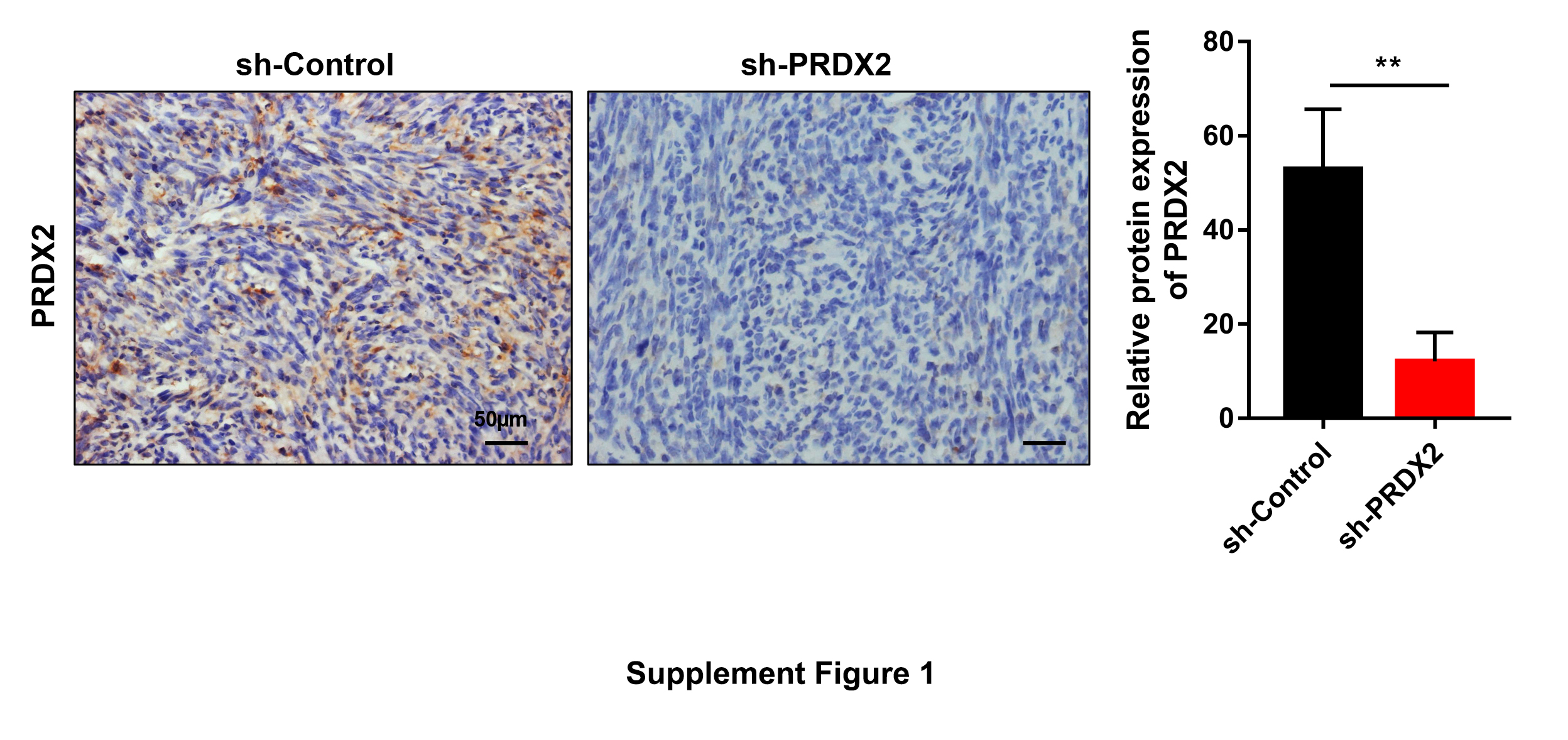

Supplement: Supplementary Materials — Figure S1: the expression of PRDX2 after knocking down PRDX2 in vivo. The protein expression of PRDX2 was significantly reduced in the sh-PRDX2 group compared with the sh-Control group by immunohistochemistry assay in vivo. [file 8359860.f1.jpg]
